# Chemotherapy-induced small bowel perforation in a patient with extrapulmonary small-cell carcinoma of the small bowel

**DOI:** 10.3747/co.v15i6.386

**Published:** 2008-12

**Authors:** S. Owen, M. Chasen

**Affiliations:** * Department of Medical Oncology, McGill University Health Centre, McGill University, Montreal, QC

Extrapulmonary small-cell carcinoma (epsmcc) is a rare disease, and involvement of the small bowel is even more rare. Overall, epsmcc accounts for only 0.1%–1% of all gastrointestinal tract (git) malignancies, which mostly affect the esophagus and colon. It is frequently metastatic at presentation, with high-grade pathologic features and aggressive clinical behaviour. Management strategies are varied, but chemotherapy remains the cornerstone of treatment. Response to platinum-based chemotherapy is between 50% and 100%, but relapses are almost universal, and prognosis is poor.

Here, we report the first case in the literature of a patient with chemotherapy-related intestinal perforation from an epsmcc of the small bowel. This patient presented with a large abdominal mass encasing the small bowel. The mass responded rapidly and considerably to chemotherapy, but the patient’s subsequent course was characterized by recurring intra-abdominal abscess formation. Current knowledge of epsmcc is reviewed, and clinical correlates on the approach to perforation of high-grade intestinal malignancies are explored.

## 1. INTRODUCTION

Extrapulmonary small cell carcinoma (epsmcc) is rare and represents a clinicopathologic entity distinct from pulmonary small-cell carcinoma. It has been described in all anatomic sites outside the central nervous system^1^, but most commonly involves the gastrointestinal tract (git), the genitourinary and reproductive systems, the salivary glands and sinuses, and lymph nodes^1^,^2^. Overall, epsmcc represents 0.1%–1% of git malignancies^3^.

Within the git, most tumours occur within the large bowel (39%), esophagus (30%), and stomach (8%); only about 3% involve the small bowel^4^. In a review of epsmcc of the duodenum in 2004, only 9 cases had been reported in the literature^5^.

Clinical presentation often involves weight loss and anorexia, or site-specific symptoms such as abdominal pain, obstruction, bleeding, or mass. Staging workup depends on the site of the primary, but often involves computed tomography (ct) imaging of the chest, abdomen, and pelvis, and git endoscopy. Diagnosis is confirmed when biopsy shows small cells with scanty cytoplasm and round nuclei combined with histologic features typical of high-grade neuroendocrine tumours. Staging can be defined by the tumour–node–metastasis (tnm) system, but more commonly uses the Veterans Administration Lung Study Group (valsg) system which identifies two distinct stages: limited stage (ls) or extensive stage (es), based on whether the disease is confined to a locoregional anatomic location or whether it is not.

The treatment approach has evolved from being primarily surgical to being centered on chemotherapy, particularly platinum-based regimens. The precise role of surgery in ls disease is not well defined, nor have any studies examined the role of surgery as prophylaxis for potential chemotherapy-induced complications such as perforation or hemorrhage.

## 2. CASE PRESENTATION

A 72-year-old man presented to our hospital with a 2-week history of lower abdominal pain, constipation, and weight loss of 7.3 kg. One month earlier, he had presented to another institution with a 1-month history of fatigue and dyspnea. On that occasion, he was found to be anemic (hemoglobin: 76 g/L), for which an endoscopy and colonoscopy were performed. Those tests failed to reveal any cause for the anemia. Physical exam demonstrated a firm, slightly tender, palpable mass in the upper abdomen, but no hepatomegaly or splenomegaly. There was also no jaundice or systemic evidence of liver disease, and no palpable lymphadenopathy. No rales, wheezes, or areas of reduced air entry were observed on auscultation. He had a performance status of 2.

**FIGURE 1:**
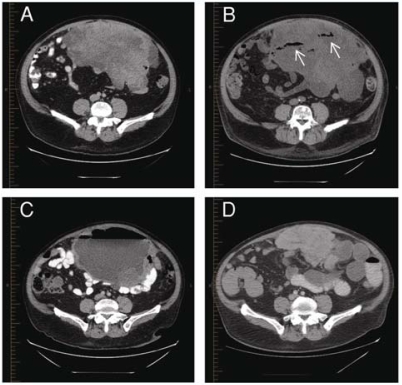
Extrapulmonary small-cell carcinoma involving the small bowel. (A) Large anterior abdominal tumour seen at initial presentation. (B) After initiation of chemotherapy, gas bubbles (arrows) indicate sites of tumour necrosis. (C) A large abscess develops from perforation of the small bowel. (D) Reduction of tumour size after 2 cycles of chemotherapy and drainage of the abscess.

Imaging of the abdomen by ct showed a large heterogeneous mass appearing to arise from the small bowel mesentery, measuring 15.7 × 26.3 × 22 cm, and partly encasing loops of small bowel [Figure 1(A)]. No adenopathy or other significant intra-abdominal pathology was observed. Imaging of the chest by ct demonstrated a single 2.2 × 2.1-cm subpleural lesion in the left lower lobe of the lung, abutting the inner aspect of the thoracic wall, appearing to arise from the abdomen, with no intrathoracic adenopathy. A ct-guided biopsy of the abdominal mass demonstrated a poorly differentiated neuroendocrine carcinoma with features of a small-cell carcinoma^a^. A bone marrow biopsy revealed no malignant cells. A bone scan and imaging of the head by ct were negative for bone and brain metastases respectively. A diagnosis of epsmcc was made, and after prompt placement of a peripherally inserted central catheter, treatment with etoposide (100 mg/m^2^, days 1 to 3) and cisplatin (80 mg/m^2^, day 1) began, with planned cycles of 21 days.

From day 1 to day 6 of chemotherapy, the patient developed recurring fever spikes (maximum: 38.8°C), but preliminary investigations did not reveal a source. On day 6, he developed worsening abdominal pain, somnolence, tachycardia, and hypotension. Peritoneal signs were present on physical exam. Imaging of the abdomen by ct showed small pockets of gas bubbles at the site of the tumour, consistent with tumour necrosis [Figure 1(B)].

Broad-spectrum antibiotics were instituted, and surgical consultation was requested. Surgical intervention was refused because of a high perioperative risk and impending neutropenia anticipated from the chemotherapy.

Over the following 4 days, the patient’s clinical picture remained tenuous. He developed acute renal failure (creatinine rose to 188 μmol/L from a baseline of about 110 μmol/L) and anasarca. Antibiotics were added, ultimately including imipenem, vancomycin, metronidazole, ciprofloxacin, and fluconazole. With these interventions, the patient’s hemodynamics and overall clinical picture normalized by day 10.

The patient recovered well from the foregoing events and received his 2nd cycle of chemotherapy on schedule. In light of the renal failure and gross edema, to which the cisplatin may have contributed because of its nephrotoxicity and the large fluid volumes given with the drug, cisplatin was replaced with carboplatin (area under the curve: 6). Antibiotics were discontinued, adequate diuresis was achieved, and the patient was discharged home on day 8 of cycle 2.

The patient returned to clinic on day 16 of cycle 2 with complaints of fatigue and dyspnea. He received 2 units of packed red blood cells because of a hemoglobin level of 85 g/L, and repeat ct imaging of the abdomen was requested to evaluate tumour response. On the patient’s return to the clinic 1 week later (day 22), his symptoms had not improved. Fever and worsening abdominal pain were now present. The abdominal ct demonstrated a large 25 × 16-cm cystic abdominal mass with air–fluid levels at the site of the previous tumour [Figure 1(C)], and leucocytosis was found (17.8 × 10^9^). The patient was sent for urgent ct-guided abdominal drainage, which removed more than 4 L purulent material, and intravenous antibiotics were administered. Symptoms improved immediately with drainage. The abdominal exudate grew *Klebsiella, Bacteroides, Enterococcus,* and *Staphylococcus aureus.*

After 24 hours of observation in the emergency room, the patient was discharged home with a prescription for 2 weeks of oral antibiotics consisting of ciprofloxacin, metronidazole, and amoxicillin with clavulanic acid. The drain was removed after 8 days. Imaging by ct on the day before drain removal showed complete resolution of the abscess and significant reduction in tumour size (Figure 1(D)].

The patient’s 3rd cycle of chemotherapy occurred on schedule and without incident, and at the time of writing, he was beginning a 4th cycle.

## 3. DISCUSSION

Like its counterpart in the lung, epsmcc has an aggressive clinical presentation and high-grade pathologic features. It is frequently metastatic at presentation, and prognosis for these tumours is poor, particularly for epsmcc of the git. In this location, the tumour tends to demonstrate es disease and high-grade histology on presentation more frequently than epsmcc of other sites does^1^,^3^. For git epsmcc, survival for untreated patients is only several weeks; it is 6–12 months for patients receiving treatment^3^. Identified prognostic factors include stage of the disease (by either the valsg or the tnm staging system), receipt of systemic treatment, and for esophageal epsmcc, age and tumour size 6.

Treatment strategies are varied and include surgical, pharmacologic, radiotherapeutic, and combined approaches. However, chemotherapy, either alone or as part of a combined approach, has emerged as the cornerstone of treatment^1^–^4^,^6^,^7^. Various drug combinations have been tried, mostly including cisplatin, etoposide, cyclophosphamide, or doxorubicin as the main agents. Response rates of 50%–100% have been reported, with the best responses seen with platinum-based regimens^3^. These response rates are high, but they are generally less than the rates seen with pulmonary small-cell carcinoma, perhaps reflecting the prevalence of mixed histology in 30%–40% of specimens^4^,^6^. Unfortunately, despite the high tumour response, almost all patients relapse and succumb; however, reports of long-term survivors have been published^8^–^10^.

In the present case, rapid response of a large, intestinal-associated epsmcc to platinum-based chemotherapy was complicated by intestinal perforation. Perforation is evidenced by the temporal correlation between chemotherapy administration, radiologic evidence of tumour necrosis, clinical manifestations of sepsis and acute abdomen, and subsequent polymicrobial intra-abdominal cultures that grew mostly enteric organisms. This sequence supports a pathophysiologic mechanism in which wall integrity of the small bowel is lost as the tumour rapidly necroses in response to chemotherapy. It is also possible, however, that the result could be related to an agent-specific risk, as is seen with bevacizumab^11^. Recurrence of the abscess during the 2nd cycle of chemotherapy suggests the need for a more definitive treatment strategy.

Bowel perforation has been extensively described in metastatic lung cancer^11^–^13^, but the present report is the first of such a case for primary small-cell carcinoma of the small bowel. Thus, no current studies are available to guide clinical management. It may therefore be useful to examine the literature of other highly chemo-responsive tumours of the git—namely, lymphomas. Perforation is a common complication in this group of diseases, frequently seen at disease presentation and portending a poor prognosis^14^,^15^. No surgical benefit is observed for gastric lymphomas, but failure-free survival is significantly correlated with resection of intestinal lymphomas^16^. However, the benefit in failure-free survival in the aforementioned study did not result from prevention of perforation, because only 1 case of bowel perforation occurred. Nevertheless, surgical resection of intestinal lymphomas before chemotherapy is currently recommended in most cases^17^.

## 4. CONCLUSIONS

Extrapleural small-cell carcinoma of the git is rare and portends a poor prognosis. Response to chemotherapy is strong and often rapid, but relapses are almost universal, with few survivors beyond 2 years. In the case reported here, high chemosensitivity resulted in treatment-related perforation of the small bowel, sepsis, and abscess recurrence during a subsequent cycle of chemotherapy. Based on the literature of git lymphomas, we suggest that surgery be considered part of the management approach in some patients with epsmcc of the small bowel. As a theoretical framework, surgery should be advised if the risk of fatal complications such as perforation or hemorrhage outweigh the surgical mortality and morbidity. Given the lack of feasibility of prospective trials for this rare entity, large retrospective analyses are needed to better characterize the risk factors for perforation among patients with epsmcc of the git and to help guide future clinical management.

## Footnotes

a Stain results: periodic acid Schiff (pas) and pas after diastase, negative; cytokeratin AE1/3 and CK8/18, positive; cytokeratin CK7, negative; cytokeratin CK20, negative; prostate-specific antigen, negative; thyroid transcription factor 1, negative; synaptophysin and neuron-specific enolase, positive; CD10 and Bcl16, negative; desmin, negative; CD45 and CD20, negative; Bcl2, positive; Ki67, 70%–80%; Fli1, negative; CD99, positive; Wilms tumour 1, negative.
